# Covalent Modification of Human Serum Albumin by the Natural Sesquiterpene Lactone Parthenolide

**DOI:** 10.3390/molecules20046211

**Published:** 2015-04-09

**Authors:** Michael Plöger, Jandirk Sendker, Klaus Langer, Thomas J. Schmidt

**Affiliations:** 1 Institute of Pharmaceutical Technology and Biopharmacy, University of Münster, PharmaCampus, Corrensstr. 48, D-48149 Münster, Germany; E-Mails: michaelploeger@googlemail.com (M.P.); k.langer@uni-muenster.de (K.L.); 2 Institute of Pharmaceutical Biology and Phytochemistry, University of Münster, PharmaCampus, Corrensstr. 48, D-48149 Münster, Germany; E-Mail: jandirk.sendker@uni-muenster.de

**Keywords:** sesquiterpene lactone, parthenolide, human serum albumin, tryptic digest, UHPLC/+ESI-QqTOF MS

## Abstract

The reactivity of parthenolide (PRT), a natural sesquiterpene lactone from *Tanacetum parthenium* (Asteraceae), with human serum albumin (HSA) was studied by UHPLC/+ESI-QqTOF MS analysis after tryptic digestion of albumin samples after incubation with this compound. It was found that the single free cysteine residue, C34, of HSA (0.6 mM) reacted readily with PRT when incubated at approximately 13-fold excess of PRT (8 mM). Time-course studies with PRT and its 11β,13-dihydro derivative at equimolar ratios of the reactants revealed that PRT under the chosen conditions reacts preferably with C34 and does so exclusively via its α-methylene-γ-lactone moiety, while the epoxide structure is not involved in the reaction.

## 1. Introduction

Sesquiterpene lactones (STLs) are a large class of natural terpenoid compounds mainly found in plants of the Asteraceae family. These compounds are well known for their wide range of biological activities. Most sesquiterpene lactones contain reactive partial structures, such as α-methylene-γ-lactone, cyclopentenone or other activated α,β-unsaturated carbonyl moieties. Many of these compounds’ biological effects have been explained by their potential to react with nucleophilic structure elements in enzymes and transcription factors, thereby modifying the biological functions of such essential biomolecules (for an overview, see [1]). Furthermore, the sensitizing potential of many STLs, leading to contact allergy, is attributed to their potential to function as haptens in type IV allergic immune reactions. This effect is explained by STLs’ reactivity as Michael acceptors, which covalently modify proteins of the skin under the formation of full allergens [2,3]. Free cysteine thiol groups are thought to be especially susceptible to Michael reaction with the STLs’ α,β-unsaturated carbonyl structures. Although a variety of examples exist where the reaction of such compounds with low molecular weight thiols, such as glutathione and free cysteine, have been studied [1,2,4,5,6,7], surprisingly little direct experimental evidence exists for the covalent modification of proteins by sesquiterpene lactones [8].

Serum albumin is the most abundant protein in human body fluids [9,10], where it accounts for about 60% of the total protein content and reaches concentrations of about 0.6–0.7 mM in blood plasma and about 0.18 mM in interstitial fluid [10]. One of the important functions of human serum albumin (HSA) is to bind low molecular weight substances, such as drugs and toxins, and, thereby, to reduce their free plasma concentration. Among various potential binding sites for such compounds, this 65-kDa protein also possesses one free thiol group (C34), which has been shown to represent a target for electrophilic xenobiotics. A variety of reports on the covalent modification of HSA are found in the literature [11,12,13,14]. In some reports, the covalent attachment of xenobiotics is intended in order to use the body’s own HSA as a drug carrier for albumin-binding prodrugs [12,13,14].

The concentration of HSA being quite high in interstitial liquid, where it has been demonstrated to be the main protein constituent [15], reaction of STLs with HSA may also be a potential source of the, yet unknown, full allergens responsible for sensitization against plants of the Asteraceae family or their pharmaceutical and cosmetic products [2,3]. 

**Figure 1:**
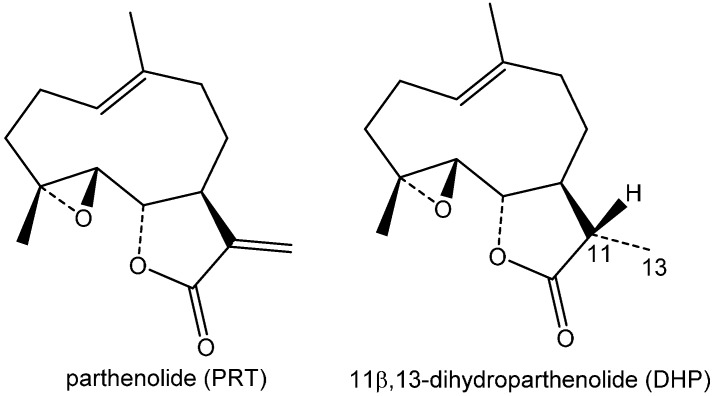
Structures of parthenolide (PRT) and 11β,13-dihydroparthenolide (DHP).

The sesquiterpene lactone parthenolide (PRT, Figure 1) is known to exhibit a variety of biological effects [16,17,18,19]. It is the major STL constituent of the medicinal plant *Tanacetum parthenium* (feverfew, family Asteraceae), which is used as an ingredient of anti-migraine preparations [20,21]. Parthenolide has also been reported to have sensitizing potential for causing allergic contact dermatitis [1,2,3,22,23,24]. We therefore chose this compound as the model STL to investigate its potential for covalent reactions with human serum albumin by UHPLC/+ESI-QqTOF MS.

## 2. Results and Discussion

The reaction of parthenolide (PRT) with human serum albumin (HSA) was investigated at a near-physiological concentration of the protein (40 mg/mL ≈ 0.6 mM) in aqueous solution (pH 8) with an approximately 13-fold molar excess of PRT. After 2 h, one aliquot of the reaction mixture was purified by gel chromatography on a PD-10 column and then lyophilized. The tryptic digestion of the lyophilized HSA-PRT-conjugate was carried out by incubation with trypsin (sequencing grade) in ammonium bicarbonate buffer (pH 7.9) for 24 h at 37 °C (Sample A). A second aliquot of the reaction mixture was directly lyophilized and submitted to trypsin digestion, *i.e.*, without prior removal of excess PRT (Sample B). For comparison, a sample of untreated HSA was submitted to tryptic digestion. Due to the fact that sesquiterpene lactones are known to react readily with free thiol groups [1,2,4,5,6,7], the tryptic digestion was performed without the usual reduction of disulfide bonds, in order to avoid reactions with such cysteine residues in HSA that are involved in disulfide linkages in the native protein.

The samples obtained by tryptic digestion from untreated HSA and from HSA treated with PRT were submitted to UHPLC/+ESIQqTOF MS analysis and compared. Peptide fragments characterized by an increase of molecular mass by 248.1413 amu, corresponding to the addition of a molecule of PRT, were searched in the chromatograms of the PRT-treated samples.

### 2.1. LC/MS-Analysis of Untreated HSA Digest

**Figure 2:**
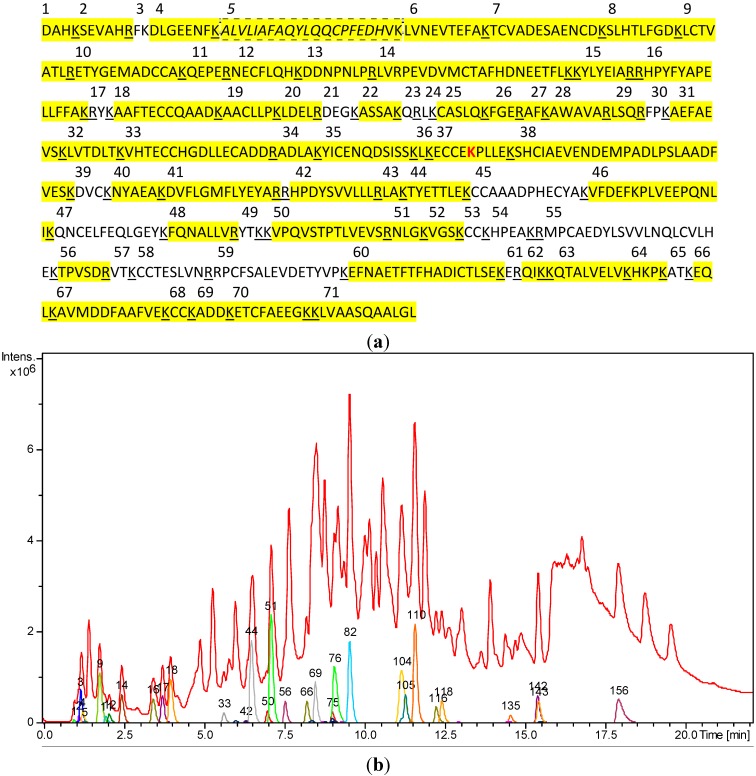
(**a**) Amino acid sequence of human serum albumin (HSA) with tryptic cleavage sites (underlined). The fragment T21-41 containing the only free cysteine residue (C34) is marked with a dashed line. Amino acids that are part of the tryptic peptides detected under our analytical conditions are marked in yellow. For fragment sequences and mass data, see Table 1. (**b**) LCMS analysis of the tryptic digest mixture obtained from untreated HSA. Red: total ion chromatogram (TIC). Colored: mass chromatograms for the various peptide fragments that could be assigned particular parts of the HSA sequence. For numbering, see Table 1; the fragment labeled 156 represents the (T21-41)_2_SS homodisulfide.

The tryptic peptide fragments of native HSA detectable under the chosen LC/MS conditions are listed in Table 1. Figure 2 shows their sequence in the primary structure and occurrence in the chromatogram. In total, fragments accounting for 471 (80.5%) of the 585 amino acids of HSA could be detected and assigned.

Of special interest is the tryptic fragment consisting of amino acids 21–41 (T21-41), since it contains the sole free cysteine residue (C34) of HSA. The singly protonated molecular ion [M+H]^+^ of T21-41 would possess a monoisotopic mass (m_i_) of 2,433.2635 amu, so that multiple protonation under positive ESI conditions would be expected to yield multiply charged ions [M+*n*H]*^n^*^+^ at *m*/*z* 1217.1354, 811.7594 and 609.0714 (*n* = 2, 3 and 4, respectively). Quite noteworthy, these ions could not be detected in the LC/MS chromatogram. Instead, a peak was detected that contained multiply-charged mass signals with monoisotopic peaks at *m*/*z* 1216.6427, 973.5146, 811.4319 and 695.6569 amu. Their charge deconvolution unambiguously showed that they represented the [M+4H]^4+^, [M+5H]^5+^, [M+6H]^6+^ and [M+7H]^7+^ species of a peptide with a monoisotopic mass m*_i_* for [M+H]^+^ of 4863.5343 amu. This mass corresponds to a dimer of T21-41 formed under the loss of two hydrogen atoms (theoretical m*_i_* for [M+H]^+^: 4,863.5190), which clearly shows that this peptide is present in the digested sample as a homo-disulfide, *i.e.*, (T21-41)_2_SS. Furthermore, the peaks of heterodisulfides of T21-41 in which C34 has reacted with cysteine (T21-41SSCys) and with homocysteine (T21-41ShCys) could be identified by their multiple protonated ions (see Table 1). It is known that only about 70% of HSA has a free C34 thiol group and that the remaining fraction exists in the form of such heterodisulfides, termed non-mercaptalbumins [25,26,27]. A glutathione-disulfide, which has also been reported, could not be detected in our samples. The absence of this species from commercial samples of HSA is in agreement with observations of other authors [27].

**Table 1:** Results of the UHPLC/+ESI-QqTOF MS analysis of the tryptic digest fragments of untreated HSA. For fragment numbering, compare Figure 2a.

Tryptic Fragment No.	Theoretical Molecular Mass of [M+H]^+^	Molecular Mass of [M+H]^+^ after Deconvolution	Deviation (mDa/ppm)	Charge States (n) of Detected Ions [M+nH]^n+^	Retention Time (min)	Compound No. (Figure 2b)
1	470.2358	470.2340	1.8/3.8	1	0.99	1
2	698.3580	698.3579	0.1/1.0	1, 2	1.13	2
3	294.1812	n.d.				
4	951.4418	951.4377	4.1/4.3	1, 2	3.72	17
5	2433.2635	n.d.				
5-SS-5	4863.5037	4863.5344	30.7/6.3	4, 5, 6, 7	17.87	156
5-SS-Cys	2552.2676	2552.2778	10.2/4.0	3, 4, 5	15.38	143
5-SS-hCys	2566.2832	2566.3146	31/12.1	3, 4, 5	9.00	75
6	1149.6150	1149.6221	7.1/6.2	1,2	7.10	51
7	1382.5195	1382.5358	16.3/11.8	1, 2	1.19	3
8	1017.5364	1017.5353	1.1/1.1	1, 2, 3	6.98	50
9 + 10 + 11 + 12	3848.7117	3848.7399	28.2/7.3	5, 6	6.32	42
13	940.4483	940.4493	1.0/1.1	1,2	1.78	9
14 + 18 + 19	4560.1368	4560.1741	37.3/8.2	4, 5, 6, 7, 8	9.53	82
15	927.4934	927.4921	1.3/1.4	1, 2	6.49	44
16	1742.8941	1742.8983	4.2/2.4	2, 3	3.43	135
17	310.1761	n.d.				
20	645.3566	645.3621	5.5/8.5	1, 2	1.96	11
21	448.2038	n.d.				
22	463.2511	463.2496	1.5/3.2	1	0.99	1
23	303.1775	n.d.				
24	260.1969	n.d.				
25 + 33	2560.0676	2560.0647	2.9/1,1	4, 5	5.64	33
26	508.2514	508.2525	1.1/2.2	1	1.25	4
27	365.2183	365.2185	0.2/0.5	1	1.19	3
28	673.3780	673.3788	0.8/1.2	1, 2	3.43	16
29	503.2936	503.2932	0.4/0.8	1	1.13	2
30	391.2340	391.2326	1.4/3.6	1	1.32	5
31	880.4411	880.4411	0/0	1, 2	2.46	14
32	789.4716	789.4691	2.5/3.2	1, 2	3.98	18
34	517.2980	517.2995	1.5/2.9	1	1.19	3
35 + 36 + 37 + 38	5730.6464	5,730.6533	6.9/1.2	4, 5, 6, 7, 8, 9	11.14	104
39	464.2173	n.d.				
40	695.3359	695.3439	8.0/11.5	1	1.19	3
41	1623.7876	1623.7938	6.2/3.8	2, 3	15.36	142
42	1311.7419	1311.7362	5.7/4.3	1, 2, 3	12.39	118
43	331.2340	331.2354	1.4/4.2	1	1.19	3
44	984.4884	984.4889	0.5/0.5	1, 2	2.07	12
45	1381.5334	n.d.				
46	2045.0954	2,045.1124	17.0/8.3	2, 3, 4	11.56	110
47	1600.7312	n.d.				
48	960.5630	960.5584	4.6/4.8	1, 2	7.54	56
49	411.2238	n.d.				
50	1511.8428	1511.8521	9.3/6.2	2, 3	8.21	66
51	431.2613	431.2608	0.5/1.2	1	1.19	3
52	390.2350	390.2300	5.0/17.2	1	0.99	1
53	353.1312	n.d.				
54	581.3052	n.d.				
55	2404.1709	n.d.				
56	674.3468	674.3529	6.1/9.0	1	1.19	3
57	347.2289	n.d.				
58	1024.4550	n.d.				
59	1853.9102	n.d.				
60 + 68 + 69 + 70 + 71	3563.5102	3,563.6537	143/40	4, 5	12.21	116
61	304.1615	n.d.				
62	388.2554	388.2550	0.4/1.0	1	1.19	3
63	1000.6037	1000.6029	0.8/0.8	1, 2	8.47	69
64	509.3194	509.3184	1.0/2.0	1	0.99	1
65	319.1976	n.d.				
66	517.2980	517.2995	1.5/2.9	1	1.19	3
67	1342.6348	1342.6417	6.9/5.1	2, 3	11.26	105
68	1013.5990	1013.6084	9.4/9.3	1, 2	9.06	76

Very noteworthy, the unmodified T21-41 fragment could not be detected at all in the tryptic digest sample, although being thoroughly sought.

The dimeric homodisulfide (T21-41)_2_SS, however, must, at least in part, have been formed by oxidation during the workup process, during or after tryptic digestion. Although HSA is known to exist in part as a dimer (see [28] and the literature cited there), the exclusive presence of the (T21-41)_2_SS fragment in the digested sample is likely to be due to the reaction of the monomeric fragment during workup. The free cysteine C34 is located in a hydrophobic crevice about 9.5 to 10Å deep [28,29] to which only low-molecular weight compounds have access. The complete artificial dimerization of HSA requires, e.g., a reaction mediated by mercury (II) and oxidation with iodine. Under such conditions, the conformation of HSA is disorganized, so that the free cysteine residues of two HSA molecules can react with each other [28]. It can hence be concluded that under the chosen conditions, the non-mercaptalbumin fraction of T21-41 is completely oxidized to the homodisulfide during the workup process, *i.e.*, after the fragment has been released from the protein. It is therefore very straightforward that in the case of a covalent modification of C34 in the intact native protein by an STL, the amount of the homodisulfide found after sample workup must be lower than that obtained from the unmodified protein. Thus, in the case that the free C34 of the native protein would react to completeness with a cysteine-modifying agent, such as PRT, this peak should be much smaller after tryptic digestion. In the case of an incomplete reaction, a direct comparison of the peak of this disulfide with that of a potential PRT-conjugate of T21-41 detectable after a certain reaction time can be expected to give valuable insight into the reactivity of PRT towards C34 of HSA.

At the same time, reaction of an STL with another amino acid within the same fragment would be likely to result in a signal for the dimer plus one STL moiety, *i.e.*, (T21-41)_2_SS + STL, and/or a dimer plus two STL molecules, (T21-41)_2_SS + 2STL. The absence of such fragments and presence of a monomer + STL signal would hence prove that the reaction has indeed occurred at C34.

### 2.2. Covalent Modification of C34 by Parthenolide

Parthenolide (PRT) was found to react readily with HSA at the free cysteine C34 in T21-41. The presence of the covalent adduct could be deduced from the occurrence of a new peak with a molecular mass corresponding to T21-41 + PRT (monoisotopic signals for [M+*n*H]*^n^*^+^, at *m*/*z* 1341.2156, 894.4849 and 671.1156 (*n* = 2, 3, 4); deconvolution result m*_i_* for *n* = 1: 2,681.4330; theoretical values: 2681.4048, 1341.2061, 894.4731, 671.1068, respectively, for *n* = 1, 2, 3, 4). Besides this covalent adduct of PRT, the dimeric disulfide (T21-41)_2_SS was still present in both samples (A and B) treated with this compound. In comparison with the unmodified protein, the peak size of (T21-41)_2_SS is diminished to about 30% and 20% in Samples A and B, respectively, so that approximately 70%–80% of the total HSA must have reacted. Since no signals for a PRT-modified (T21-41)_2_SS + PRT or (T21-41)_2_SS + 2PRT were detectable (although thoroughly sought), the reaction must have occurred at C34 as expected. 

**Figure 3:**
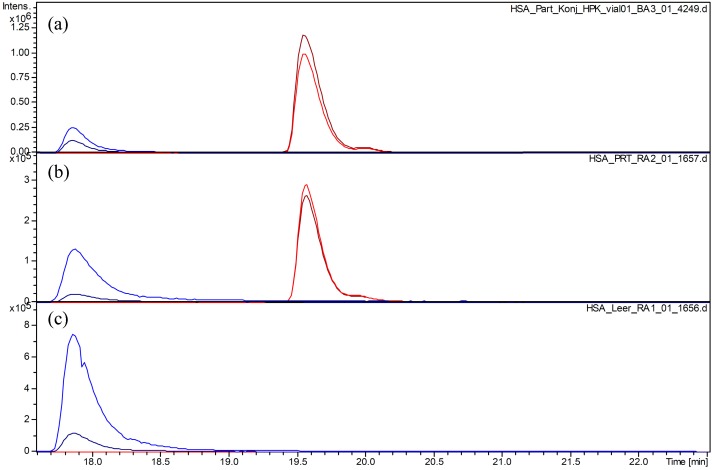
Extracted ion chromatograms for the (T21-41)_2_SS homodisulfide (blue plots) and for the monomeric (T21-41) + parthenolide (PRT) fragment (red plots). (**a**) HSA + PRT, Sample A; (**b**) HSA + PRT, Sample B; (**c**) HSA without PRT treatment. Extracted ion chromatograms for (T21-41)_2_SS (*m*/*z* 811.8 [M+6H]^6+^, blue; 973.5 [M+5H]^5+^, dark blue) and for T21-41 + PRT (*m*/*z* 671.1 [M+4H]^4+^, dark red; 894.5 [M+3H]^3+^, red).

Figure 3 shows a plot of extracted ion chromatograms for T21-41 + PRT and (T21-41)_2_SS in Samples A and B. The relative size of the peaks is very similar in these two samples, indicating that no further reaction of PRT with T21-41 occurred during or after tryptic release of this fragment in Sample B, from which excess STL was not removed prior to digestion. This shows that the reaction under these conditions, at a 13-fold excess of PRT, has already reached equilibrium after 2 h.

### 2.3. Time Course of the Reaction between Parthenolide and HSA

**Figure 4:**
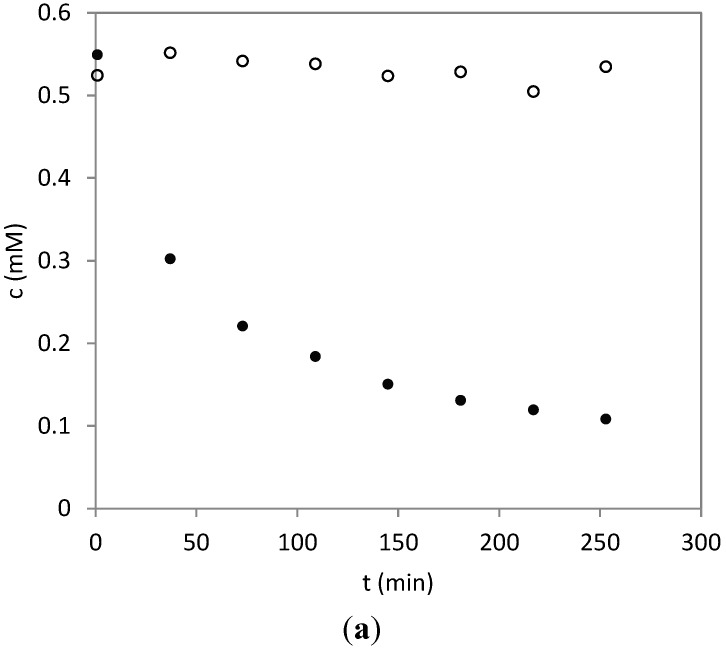

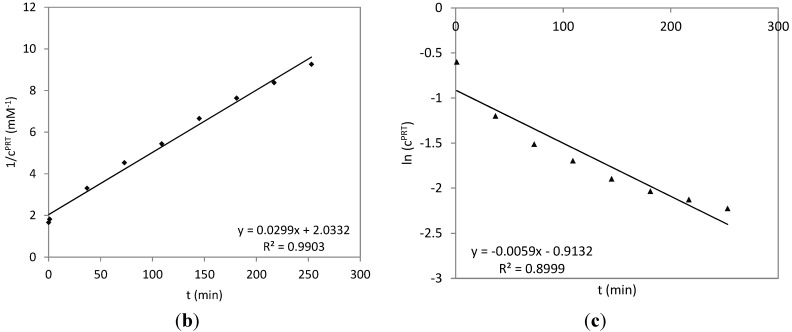
Time course of the reaction between HSA and PRT (filled circles) and for 11β,13-dihydroparthenolide (DHP, open circles); both sesquiterpene lactones (STLs) were incubated with HSA at an equimolar concentration (1:1, 0.6 mM). (**a**) The concentration of PRT and DHP *vs.* the reaction time; (**b**) linearization of the PRT time course according to the second order rate law; (**c**) linearization of the PRT time course according to the first order rate law. The reaction is more likely to proceed by second-order than by (pseudo) first-order kinetics, so that HSA can be assumed to represent one reactive site per molecule rather than multiple sites. From the data of DHP in (a), it becomes obvious that the α-methylene-γ-butyrolactone moiety is the only structure element in PRT responsible for the reaction with HSA.

In order to obtain information about the extent and rate of reaction between HSA and PRT, time course experiments were conducted by UHPLC/+ESI-QqTOF MS. The reaction was monitored at an equimolar ratio of the two reactants (0.6 mM) by repeated analysis of a sample incubated at pH 8.2 at 25 °C. In the resulting chromatograms, the peak area of PRT was integrated based on extracted ion chromatograms for the prominent fragment ion at *m*/*z* 231 ([M+H-H_2_O]^+^). Figure 4a shows a diagram of the PRT concentration over time. The reaction even at this concentration of PRT proceeds quite quickly with a half-life of about 37 min. Plots of either 1/c^PRT^ or ln(c^PRT^) *vs.* time (Figure 4b,c, respectively) show that the reaction follows a second order rate law, since the former plot yields a linear trend line with R^2^ = 0.99, while the latter shows a stronger curvature and an R^2^ < 0.9. The presence of a second order reaction under these equimolar conditions corroborates the hypothesis that HSA is characterized by one primary reactive site for PRT addition, *i.e.*, one highly reactive site exists per HSA molecule rather than multiple equally-susceptible sites. In this latter case, an excess of reactive sites over PRT molecules would be expected to lead to a more significant deviation from second order kinetics under these conditions and would more likely appear pseudo first order. This finding therefore indicates that PRT, when incubated at an equimolar concentration with HSA, reacts with the protein in a ratio of 1:1, which confirms the observation from the MS-experiment that it selectively binds to C34.

Since PRT itself contains two potentially reactive sites, an α-methylene-γ-lactone and an epoxide function, an analogous experiment was conducted with 11β,13-dihydroparthenolide (DHP), whose structure contains only the latter. The resulting time course data are plotted in Figure 4a along with those of PRT. It is obvious that DHP does not react with HSA to any significant extent under identical conditions, so that the α-methylene-γ-lactone structure element is solely responsible for the reaction of PRT with HSA, and the epoxide moiety does not play any role.

## 3. Experimental Section

### 3.1. Investigated Compounds

Parthenolide (PRT) was purchased from AppliChem (Darmstadt, Germany; Batch 9L003782). 11β,13-dihydroparthenolide (DHP) was a gift from N. H. Fischer (Denton, TX, USA). The identity and purity was determined by HPLC and/or NMR analyses; the purity was >90% in both cases.

Human serum albumin (Fraction V, purity 96%–99%, batch: 049K7535) and trypsin (sequencing grade) were obtained from Sigma-Aldrich (Steinheim, Germany) and PD-10 columns (Sephadex G-25M) from GE-Healthcare (Buckinghamshire, UK). All other reagents were purchased from Roth (Karlsruhe, Germany). All chemicals were of analytical grade and used as received.

### 3.2. Sample Preparation

#### 3.2.1. Preparation of Parthenolide-HSA Conjugate

HSA (50 mg) was dissolved in 1.0 mL purified water and adjusted with 0.1 N sodium hydroxide to pH 8.2. After the addition of 250 µL of an ethanolic solution of parthenolide (10 mg/mL), the mixture (0.6 mM HSA, 8 mM PRT) was incubated for 2 h under permanent stirring (200 rpm). After incubation, the conjugate was purified from the reaction mixture using a PD-10 column according to the manufacturer’s instruction sheet with water as the eluent. Fractions 4, 5 and 6 were collected and lyophilized, yielding Sample A.

Direct lyophilization without purification of the reaction mixture after 2 h of incubation yielded Sample B.

#### 3.2.2. Tryptic Digestion

Trypsin (14 µg, sequencing grade) was added to a solution of 2 mg purified PRT-HSA conjugate (Sample A) or 2 mg of the lyophilisate of the unpurified reaction mixture (Sample B) in 0.5 mL 50 mM ammonium bicarbonate buffer at pH 7.9. The solution was incubated for 24 h under permanent shaking (1000 rpm) at 37 °C using a Thermomixer^®^ comfort device (Eppendorf, Hamburg, Germany). The hydrolysis was stopped by freezing the sample. The tryptic digestion of unreacted HSA was carried out in the same manner.

### 3.3. High-Performance Liquid Chromatography-Electrospray Ionization Mass Spectrometry

All analyses were performed with a micrOTOF-QII mass spectrometer (Bruker Daltonics, Bremen, Germany) coupled to an Ultimate 3000 RS (Dionex, Idstein, Germany) UHPLC system with a diode array detector (DAD). Separations were achieved with a Biobasic-18 column (C18, 2.1 × 150 mm, 5 µm; ThermoFisher Scientific, Schwerte, Germany) using acetonitrile:water (both containing 0.1% formic acid) for elution in a gradient from 9:1 to 4:6 in 25 min at a flow rate of 0.4 mL/min. Mass spectra were acquired in the +ESI mode in the *m*/*z* range from 50 to 3000 at a sampling rate of 2 Hz. Internal calibration of each analysis was performed by direct infusion of 20.0 µL of a 5 mM sodium formate solution at the end of the run.

### 3.4. Data Analysis

MS data were analyzed with the Data Analysis software (Bruker Daltonics, Bremen, Germany).

Calculation of the tryptic fragments’ molecular masses was performed with the web-based tool, [30].

### 3.5. Time Course of the Reaction of HSA with PRT and DHP

For the time course measurements, HSA was dissolved in the same concentration as above and mixed with an equimolar amount of PRT (both 0.6 mM) in an HPLC vial. The reaction mixture was then stored in the autosampler at 25 °C and continuously analyzed after defined time intervals. The peak areas of PRT, determined by integration of the peak in extracted ion chromatograms at *m*/*z* 231 ([M+H-H_2_O]^+^), were transformed to concentrations by direct comparison with the corresponding peak in a chromatogram obtained with an amount of PRT representing the starting concentration (0.6 mM). An analogous experiment was conducted with the same concentration of DHP. The resulting concentrations were plotted *vs.* time to yield the plot shown in Figure 4a.

## 4. Conclusions

The interaction of the sesquiterpene lactones, parthenolide (PRT) and dihydroparthenolide (DHP), with the most abundant plasma protein, human serum albumin (HSA), was analyzed by MS analysis. An extensive protein binding of PRT by the α-methylene-γ-lactone structure element to the reactive thiol group of HSA at its C34 position could be shown. To the best of our knowledge, this is the first study giving a direct experimental proof for a covalent modification of C34 in the primary structure of HSA by the α,β-unsaturated structure of PRT or any other sesquiterpene lactone. This observation adds a new interesting aspect to the numerous reports on the sensitizing potential of sesquiterpene lactones. It can be hypothesized on the background of this result that covalent modification of HSA at C34 may represent a major source of allergens responsible for hypersensitivity against certain STLs, such as PRT. This hypothesis will have to be investigated in further studies.
